# First complete mitochondrial genome from family Moinidae, *Moina macrocopa* (Straus, 1820) (Cladocera; Moinidae)

**DOI:** 10.1080/23802359.2022.2080024

**Published:** 2022-06-10

**Authors:** Sang-Eun Nam, Jaehee Kim, Jae-Sung Rhee

**Affiliations:** a Department of Marine Science, College of Natural Sciences, Incheon National University, Incheon, South Korea; b Research Institute of Basic Sciences, Incheon National University, Incheon, South Korea; c Yellow Sea Research Institute, Incheon, South Korea

**Keywords:** Complete mitogenome, Cladocera, Moinidae, *Moina macrocopa*

## Abstract

We sequenced and annotated the complete mitochondrial genome for the freshwater water flea *Moina macrocopa* (Straus, 1820). This is the first mitogenome for the family Moinidae. The complete mitogenome of *M. macrocopa* is 16,072 bp, with 35.8% A, 17.6% C, 12.8% G, and 33.8% T. The mitogenome comprises 13 protein-coding genes (PCGs), two ribosomal RNA (rRNA) genes, 22 transfer RNA (tRNA) genes, and a non-coding region. Phylogenomic analysis based on 28 in-group taxa belonging to the orders Anostraca, Diplostraca, and Notostraca is congruent with published phylogenetic relationship for cladocerans, with *M. macrocopa* being grouped with members of the Daphniidae. This mitogenome resource will be useful for future phylogenetic studies of water fleas.

Cladocerans, generally known as filter-feeding water fleas, are important zooplankton components in the food web of aquatic ecosystems because of the grazing activity of phytoplankton and because they are crucial species at higher trophic levels. The species richness, abundance, and structural and ecological diversity of cladocerans have been investigated in some detail (De Bie et al. 2007; Forró et al. 2008; Stollewerk 2010; Miner et al. 2012; Cornetti et al. 2019). They have a wide distribution, predominantly in freshwater ecosystems. Four major orders have been classically recognized in cladocerans: Anomopoda (nine families, 51 genera), Ctenopoda (two families, six genera), Onychopoda (three families, nine genera), and the monotypic Haplopoda (one family, one genus) (Forró et al. 2008). The interesting characteristics of cladoceran reproduction (i.e. cyclical parthenogenesis and sexually produced diapausing eggs) have been widely highlighted in diverse research areas (Forró et al. 2008).

The genus *Moina* Baird (Cladocera: Moinidae) is a dominant cladoceran among the zooplankton communities in freshwater ecosystems. Several species of *Moina* have been used as food sources in aquaculture and have been studied concerning physiology (Smirnov 2014; Gama-Flores et al. 2015) and toxicology (Wong et al. 1995; Oh and Choi 2012; Jia et al. 2018; Hu et al. 2020; Kim and Rhee 2021). However, exploration of phylogenetic relationships and genetic distance of moinid taxa has remained poorly studied compared with the established evolutionary history of other cladocerans due to morphological variability, incomplete taxonomy, and the lack of genomic data (Petrusek et al. 2004). Recently, based on partial sequences of genomic markers that include cytochrome c oxidase subunit I (*COI*) gene and nuclear internal transcribed spacer-1 (ITS-1), comprehensive molecular relationships of the genus *Moina* have been established in North Eurasia (Bekker et al. 2016), China (Ni et al. 2019), and Japan (Makino et al. 2020) species. Whole mitochondrial genome information is invaluable for estimating the phylogenetic relationships of cryptic species in invertebrates. In Cladocera, 13 complete mitogenomes have been sequenced from four cladoceran families and lodged at GenBank. However, there are no complete mitogenomes available for the family Moinidae.

In this study, the entire mitogenome was sequenced from a Thailand strain of *Moina macrocopa* (Straus, 1820), which was originally collected from Suphan Buri Province (14°46′N, 99°51′E; Thailand) at 2014. Specimens were transported through commercial aquaria for fish feeding in 2014 and cultured in an automated aquaculture system at Incheon National University (Incheon, South Korea). All animal handling and experimental procedures were approved by the Animal Welfare Ethical Committee and the Animal Experimental Ethics Committee of the Incheon National University (Incheon, South Korea). The specimens and DNA were deposited at the Research Institute of Basic Sciences of Incheon National University (specimen ID: 2014-cladoceran-09; https://www.inu.ac.kr/user/indexMain.do?siteId=ribs) by Dr. Sang-Eun Nam (se_nam2@inu.ac.kr). Genomic DNA was isolated from the whole body of a single individual using a DNeasy Blood and Tissue kit (Qiagen, Hilden, Germany) according to the manufacturer’s instructions. Next-generation sequencing was performed to obtain a circular mitogenome with HiSeq platform (150 bp; HiSeq X ten; Illumina, San Diego, CA) using previously described protocols (Park et al. 2020). After the quality check, 26,577,426 filtered reads were obtained from 41,007,846 raw reads. Thereafter, *de novo* assembly was conducted with various k-mers using NOVOPlasty (Dierckxsens et al. 2017) to obtain a circular contig of the *M. macrocopa* mitogenome. Mean coverage and sequencing depth were 100% and 1080.18, respectively. The resulting contig consensus sequence was annotated using MITOS2 (Bernt et al. 2013) and tRNAscan-SE 2.0 (Lowe and Eddy 1997). BLAST searches (http://blast.ncbi.nlm.nih.gov) and multiple alignment with cladoceran mitogenomes available from GenBank to confirm identity and annotation of these genes.

The complete mitogenome of *M. macrocopa* is 16,072 bp in length (GenBank accession no. MZ982313) with the typical composition, consisting of 13 protein-coding genes (PCGs), 22 transfer RNAs (tRNAs), two ribosomal RNAs (rRNAs), and a large intergenic region presumed to be the control region. The nucleotide composition was highly biased toward A + T nucleotides (69.6%), with percentages of A, T, C, and G being 35.8%, 33.8%, 17.6%, and 12.8%, respectively. The *COI* sequence showed the highest similarity (99.7%) to the *COI* sequence of *M. macrocopa* previously registered in GenBank (657 bp; GenBank accession no. KX168581). The overall genomic architecture of *M. macrocopa* mitochondria was similar to that of the previously published cladoceran mitogenomes. We constructed the phylogenetic topology of 33 members belonging to the order Anostraca, Diplostraca, and Notostraca using the concatenated set of 13 PCG sequences, with three species as an outgroup (Figure 1). Of the 12 Diplostraca species, *M. macrocopa* is closely related to *Bosmina fatalis* (Bosminidae) and *Ovalona pulchella* (Chydoridae).

**Figure 1.**
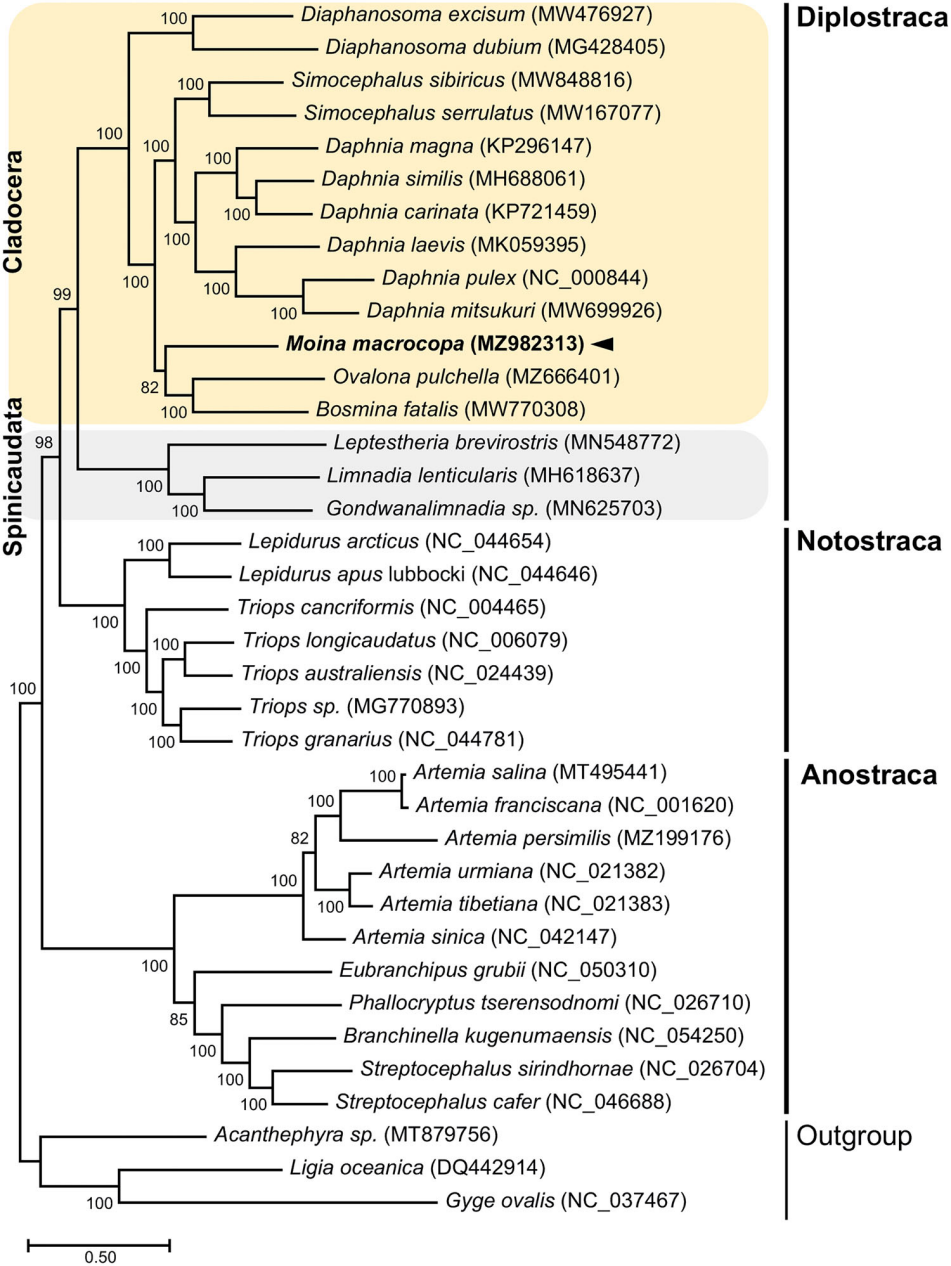
Maximum-likelihood (ML) phylogeny of 34 published mitogenomes from the Order Anostraca, Diplostraca, or Notostraca including *M. macrocopa* and three species as an outgroup based on the concatenated nucleotide sequences of protein-coding genes (PCGs). The phylogenetic analysis was performed using the maximum-likelihood method, GTR + G+I model with a bootstrap of 1000 replicates. Numbers on the branches indicate ML bootstrap percentages. DDBJ/EMBL/GenBank accession numbers for published sequences are incorporated. The black triangle means the water flea analyzed in this study.

## Author contributions

Sang-Eun Nam: conceptualization, methodology, software, and writing; Jaehee Kim: methodology, software, data curation, and writing; J.-S. Rhee: conceptualization, supervision, reviewing, and editing.

## Data Availability

The BioProject, BioSample, and SRA accession numbers are https://www.ncbi.nlm.nih.gov/bioproject/PRJNA762149, https://www.ncbi.nlm.nih.gov/biosample/SAMN21378320, and https://www.ncbi.nlm.nih.gov/sra/SRR15840885, respectively. The data that support the findings of this study are available at the National Center for Biotechnology Information (NCBI) at https://www.ncbi.nlm.nih.gov (accession number MZ982313).
